# Temporal changes in the prevalence and associates of diabetes-related lower extremity amputations in patients with type 2 diabetes: the Fremantle Diabetes Study

**DOI:** 10.1186/s12933-015-0315-z

**Published:** 2015-12-18

**Authors:** Mendel Baba, Wendy A. Davis, Paul E. Norman, Timothy M. E. Davis

**Affiliations:** School of Medicine and Pharmacology, University of Western Australia, Fremantle Hospital, PO Box 480, Fremantle, WA 6959 Australia; Podiatric Medicine Unit, University of Western Australia, Crawley, Perth, WA Australia; School of Surgery, University of Western Australia, Fremantle Hospital, Fremantle, WA Australia

**Keywords:** Type 2 diabetes, Lower extremity amputation, Risk factors, Temporal changes

## Abstract

**Background:**

To determine temporal changes in the prevalence and associates of lower extremity amputation (LEA) complicating type 2 diabetes.

**Methods:**

Baseline data from the longitudinal observational Fremantle Diabetes Study (FDS) relating to LEA and its risk factors collected from 1296 patients recruited to FDS Phase 1 (FDS1) from 1993 to 1996 and from 1509 patients recruited to FDS Phase 2 (FDS2) from 2008 to 2011 were analysed. Multiple logistic regression was used to determine associates of prevalent LEA in individual and pooled phases. Generalised linear modelling was used to examine whether diabetes related LEA prevalence and its associates had changed between Phases.

**Results:**

There were 15 diabetes-related LEAs at baseline in FDS1 (1.2 %) and 15 in FDS2 (1.0 %; *P* = 0.22 after age, sex and race/ethnicity adjustment). In multivariable analysis, independent associates of a baseline LEA in FDS1 were a history of vascular bypass surgery or revascularisation, urinary albumin:creatinine ratio, peripheral sensory neuropathy and cerebrovascular disease (*P* ≤ 0.035). In FDS2, prevalent LEA was independently associated with a history of vascular bypass surgery or revascularisation, past hospitalisation for/current foot ulcer and fasting serum glucose (*P* ≤ 0.001). In pooled analyses, a history of vascular bypass or revascularisation, past hospitalisation for/current foot ulcer at baseline, urinary albumin:creatinine ratio (*P* < 0.001), as well as FDS Phase as a binary variable [odds ratio (95 % confidence interval): 0.28 (0.09–0.84) for FDS2 vs FDS1, *P* = 0.023] were associated with a lower risk of LEA at study entry.

**Conclusions:**

The risk of prevalent LEA in two cohorts of patients with type 2 diabetes from the same Australian community fell by 72 % over a 15-year period after adjustment for important between-group differences in diabetes-related and other variables. This improvement reflects primary care foot health-related initiatives introduced between Phases, and should have important individual and societal benefits against a background of a progressively increasing diabetes burden.

## Background

There is evidence that the rates of chronic complications and death associated with diabetes are declining in developed countries [1–4], although the burden of disease remains high due to a progressive increase in diabetes prevalence [5]. In the case of lower extremity amputation (LEA), the development of multidisciplinary foot clinics and streamlined care pathways in local secondary and tertiary health care settings has been associated with significant reductions in the rates of LEA [6–9]. Most larger population-based studies have also shown a reduction in the rates of this complication [3, 10–13], but some have shown no change [14–16] or an increase [17].

Interpretation of the results of clinic- and population-based studies is, however, complicated by limitations such as use of selected patient samples, a restricted range of explanatory and confounding variables, and lack of interpretation of the findings in the light of changes in management that could have an impact on foot health. There is, therefore, the need for an assessment of temporal changes in LEA rates in community-based patient groups and in health care systems, both of which are well-characterised. The aims of the present study were, therefore, (1) to determine whether the prevalence of LEA has changed in comprehensively assessed patients with type 2 diabetes resident in a large urban Australian population in the 15 years between 1993–1996 and 2008–2011, and (2) to assess the relationship between any changes in established risk factors for LEA and its prevalence over the same period.

## Methods

### Patients and approvals

We studied participants in the Fremantle Diabetes Study Phase 1 (FDS1) and Phase 2 (FDS2) [18]. Both Phases are longitudinal observational studies carried out in the same postcode-defined geographical area surrounding the port city of Fremantle in the state of Western Australia (WA). Details of recruitment, sample characteristics including classification of diabetes type, and non-recruited patients have been published previously [18]. In brief, any patient resident in the study catchment area with a clinician-verified diagnosis of diabetes was eligible. Sources of identification and/or diagnostic data included public hospital inpatient/outpatient clinic lists and laboratory databases, notifications by local primary care/specialist physicians and allied health services including diabetes education, dietetics and podiatry, advertisements in pharmacies and local media, and word of mouth. The FDS1 protocol was approved by the Human Rights Committee, Fremantle Hospital and FDS2 was approved by the Human Research Ethics Committee of the Southern Metropolitan Area Health Service. All subjects in both Phases gave written informed consent.

We identified 2258 eligible FDS1 participants during the three-year period between 1993 and 1996 in the local population of approximately 120,000 (crude diabetes prevalence 1.9 %) and recruited 1426 (63 %). In the case of FDS2, 4639 diabetic patients were identified over the same time period between 2008 and 2011 from a population of 157,000 (crude prevalence 3.0 %) and 1668 (36 %) were recruited, including 326 surviving FDS1 participants. For FDS1, 1296 (90.9 %) of the recruited cohort had type 2 diabetes and, for FDS2, the equivalent figure was 1509 (90.4 %).

### Overview of assessment procedures

Each FDS1 participant was assessed in detail at baseline and invited to attend annual reviews for ≥5 years. For FDS2, comprehensive baseline assessments are followed by face-to-face assessments biennially rather than annually, with questionnaire follow-up in alternate years. All FDS face-to-face assessments comprise a comprehensive questionnaire, physical examination and standard fasting biochemical tests [18]. The focus of the present study was the identification of any changes in diabetes-related LEA prevalence and associates over the 15 years between recruitment periods. For this reason, baseline rather than serial data from the two Phases were analysed.

### Data collection

For both Phases, diabetes type was assessed from diabetes treatment history, BMI, age at diagnosis, nature of first presentation, and/or self-identification, and case records were consulted for evidence of ketonaemia, as well as islet autoantibodies, serum insulin and C-peptide levels, if available. Ethnic background was assessed from self-selection, country/countries of birth and parents’ birth, language(s) spoken at home and, for FDS2, country of grandparents’ birth.

In the case of foot assessment, a trained nurse performed (1) palpation of the pedal pulses (dorsalis pedis and posterior tibial), (2) measurement of the ankle brachial index (ABI), (3) general foot inspection to detect ulceration (defined, for the purposes of the present study, as located at or below the level of the malleoli), deformity, corns or callus, skin fissures, infections and nail pathology) and (4) assessment of peripheral sensory neuropathy (PSN) using the clinical features of the Michigan Neuropathy Screening Instrument (MNSI) [19]. PSN was defined as a score of >2/8 on the clinical portion of the MNSI. The patient was asked about prior foot ulceration and intermittent claudication was ascertained by determining whether pain in the calves came on during walking, caused the patient to slow down or stop, and resolved with rest. Peripheral arterial disease (PAD) was considered present if the ABI was ≤0.90 on either leg or a diabetes-related amputation (attributable to PAD) was present [20]. A major amputation was defined as through, or proximal to, the tarsometatarsal joint, and a minor one as distal to this joint [21].

Other chronic complications were ascertained using standard criteria. Self-reported stroke and transient ischemic attack were amalgamated with prior hospitalisations to define baseline cerebrovascular disease status. Patients were considered to have coronary artery disease if there was a self-reported history of, or hospitalisation for, myocardial infarction, angina, coronary artery bypass grafting or angioplasty. A subject was considered to have retinopathy if any grade of retinopathy, including maculopathy, was detected by direct and/or indirect ophthalmoscopy in one or both eyes and/or on more detailed assessment by an ophthalmologist in FDS1 or from retinal photography using a non-mydriatic camera in FDS2. The estimated glomerular filtration rate (eGFR) was calculated using the chronic kidney disease epidemiology collaboration equation [22]. Details of prior hospitalisations accessed through the Western Australian Data Linkage System [23] provided important supplementary data for ascertainment of coronary heart disease, cerebrovascular disease, foot ulceration, peripheral revascularisation and arterial bypass procedures. Coding error rates and missing patient numbers are low (around 1 % or less) for vascular complications [24].

Biochemical testing in both Phases of the FDS was carried out in the same nationally accredited diagnostic biochemistry laboratory. Between-run imprecision for all methods was <3.5 %, except for urine albumin and serum HDL-cholesterol in FDS2, for which it was <5.0 %. Serum LDL-cholesterol was estimated using the Friedewald equation. For assays that had changed between 1993 and the present, calibration equations were applied to standardize all concentrations to current assays used for FDS2 [25].

### Statistical analysis

The computer package IBM SPSS Statistics 21 (IBM Corporation, Somers, NY, US) was used for statistical analysis. Data are presented as proportions, mean ± SD, geometric mean (SD range), or, median and interquartile range (IQR) in the case of variables that do not conform to the normal or log-normal distribution. For independent samples, two way comparisons for proportions were performed by Fisher’s exact test, Student’s *t* test for normally distributed variables, and the Mann–Whitney *U*-test for variables that were not normally distributed. A two-tailed *P*-value of <0.05 was considered significant. Multiple logistic regression with forward conditional entry (*P* < 0.05 for entry, *P* > 0.05 for removal) was used to determine independent associates of prevalent LEA, with all clinically plausible variables *P* < 0.05 considered for entry into the model, except for PAD because all patients with amputation at baseline were defined as having PAD after examination of medical records. Generalized linear modelling with adjustment for age, sex and ethnicity was used to determine whether baseline associates had changed between Phases.

## Results

### Patient characteristics

Demographic, socioeconomic, anthropometric and diabetes-specific details of participants with type 2 diabetes recruited to the two Phases are summarized in Table 1. The between-phase differences in cohort characteristics have been describe elsewhere [26]. In brief, in FDS2 vs FDS1 there was a greater proportion of Aborigines, diabetes diagnosis was at a younger age, diabetes duration was longer, more were overweight/obese, and alcohol consumption was higher but more were current smokers. Indices of glycaemic control were lower in FDS2 subjects who were more likely to be insulin-treated, and systolic blood pressure and serum lipid profiles were better consistent with more intensive antihypertensive and lipid-lowering therapy. Although fewer FDS2 patients had microalbuminuria and an eGFR <60 mL/min/1.73 m^2^, they were more likely to have retinopathy and neuropathy. They were, however, less likely to have intermittent claudication and PAD against a background of more frequent prior vascular bypass surgery or peripheral revascularisation procedures.

**Table 1:** Baseline characteristics of Fremantle Diabetes Study Phase 1 (FDS1) and 2 (FDS2) participants with type 2 diabetes

	FDS1	FDS2	Difference (95 % CI)	*P*-value*
Number	1296	1509		
Age (years)	64.0 ± 11.3	65.4 ± 11.7	1.4 (0.6–2.3)	<0.001
Sex (% male)	48.6	51.8	3.2 (−0.5 to 6.9)	0.032
Ethnic background (%)				
Anglo-Celt	61.4	52.6	−8.9 (−12.5 to −5.2)	<0.001
Southern European	17.7	12.9	−4.9 (−7.6 to −2.2)	<0.001
Other European	8.5	7.4	−1.1 (−0.9 to 3.1)	0.20
Asian	3.4	4.3	0.9 (−0.5 to 2.3)	0.14
Aboriginal	1.5	7.1	5.6 (4.2–7.1)	<0.00
Other	7.5	15.8	8.4 (6.0–10.7)	<0.001
Age at diabetes diagnosis (years)	57.9 ± 11.7	55.6 ± 12.4	−2.3 (−3.2 to 1.4)	<0.001
Duration of diabetes (years)	4.0 (1.0–9.0)	8.0 (2.7–15.4)	3.7 (3.2–4.3)	<0.001
Education beyond primary level (%)	74.0	86.8	12.8 (9.8–15.7)	<0.001
Paid employment (%)	17.5	31.0	13.6 (10.4–16.7)	<0.001
Married/de facto relationship (%)	65.7	62.7	−3.0 (−6.5 to 0.6)	0.79
Alcohol use (standard drinks/day)	0 (0–0.8)	0.1 (0–1.2)	0.2 (0.1–0.4)	0.003
Smoking status (%)				
Never	44.7	45.4	0.7 (−3.0 to 4.4)	0.054
Ex-	40.2	43.9	3.6 (−0.03 to 7.3)	0.29
Current	15.1	10.7	−4.5 (−7.0 to −2.0)	<0.001
BMI (kg/m^2^)	29.6 ± 5.4	31.3 ± 6.1	1.7 (1.3–2.1)	<0.001
Obese by waist circumference (%)^a^	64.5	70.9	6.4 (2.9–9.9)	<0.001
Overweight/obese by waist:hip ratio (%)^b^	74.2	82.7	8.5 (5.4–11.6)	<0.001
Fasting serum glucose (mmol/L)	8.0 (6.5–10.3)	7.2 (6.2–8.9)	−0.8 (−1.0 to −0.6)	<0.001
HbA_1c_ (%)	7.2 (6.2–8.5)	6.8 (6.2–7.7)	−0.3 (−0.4 to −0.2)	<0.001
HbA1c (mmol/mol)	55 (44–69)	51 (44–61)	−3.5 (−4.8 to −2.1)	<0.001
Diabetes treatment (%)				
Diet	31.9	24.6	−7.3 (−10.7 to −4.0)	<0.001
Oral agents	56.0	53.4	−2.6 (−6.3 to 1.1)	0.075
Insulin ± oral agents	12.1	22.0	9.9 (7.2–12.7)	<0.001
Systolic blood pressure (mmHg)	151 ± 24	146 ± 22	−5.1 (−6.8 to −3.4)	<0.001
Diastolic blood pressure (mmHg)	80 ± 11	80 ± 12	−0.3 (−1.1 to 0.6)	0.66
On antihypertensive therapy (%)	50.9	72.6	21.7 (18.2–25.2)	<0.001
On renin-angiotensin blockers (%)	21.8	64.3	42.4 (39.1–45.8)	<0.001
Total serum cholesterol (mmol/L)	5.5 ± 1.1	4.4 ± 1.1	−1.1 (−1.2 to −1.0)	<0.001
Serum HDL cholesterol (mmol/L)	1.06 ± 0.3	1.24 ± 0.3	0.18 (0.15–0.20)	<0.001
Serum LDL cholesterol (mmol/L)	3.3 ± 0.91	2.3 ± 0.9	−1.0 (−1.1 to −0.9)	<0.001
Serum triglycerides (mmol/L)	2.2 (1.2–3.9)	1.5 (0.9–2.5)	−0.9 (−1.0 to −0.7)	<0.001
On lipid modifying treatment (%)	10.5	67.5	56.9 (54.0–59.8)	<0.001
Taking aspirin (%)	22.0	36.6	14.6 (11.3–17.9)	<0.001
Microalbuminuria or worse (%)	56.4	40.0	−16.4 (−20.0 to −12.7)	<0.001
eGFR <60 mL/min/1.73 m^2^ (%)	24.2	16.5	–7.8 (−10.8 to −4.8)	<0.001
Any retinopathy (%)	16.4	22.4	6.0 (3.0–9.1)	0.001
Peripheral sensory neuropathy (%)	30.8	58.2	27.4 (23.9–31.0)	<0.001
Ischaemic heart disease (%)	29.6	27.8	−1.8 (−5.1 to 1.6)	0.32
Cerebrovascular disease (%)	10.0	8.5	−1.4 (−3.6 to 0.8)	0.21
Peripheral arterial disease (%)	29.3	22.6	−6.7 (−10.0 to −3.5)	<0.001
Intermittent claudication (%)	14.0	9.2	−4.8 (−7.2 to −2.4)	<0.001
Arterial bypass/revascularisation (%)	1.3	3.0	1.7 (0.7–2.8)	0.013
Past hospitalisation for/current foot ulcer	1.6	2.8	1.2 (0.1–2.3)	0.17
Diabetes-related LEA (%)	1.2	1.0	−0.2 (−0.9 to 0.6)	0.22
Diabetes-related major LEA (%)	0.6	0.3	−0.3 (−0.8 to 0.2)	0.07
Diabetes-related minor LEA (%)	0.5	0.7	0.1 (−0.5 to 0.7)	0.90

Data are proportions, mean ± SD, geometric mean (SD range), median (IQR) or mean difference (95 % CI)

^a^Waist circumference ≥102.0 cm males, ≥88.0 cm females

^b^Waist:hip ratio ≥0.95 males, ≥0.80 females

* Age-, sex- and ethnicity-adjusted for interaction between Phases

### Baseline diabetes related amputation prevalence and associates

At baseline, 15 patients had undergone prior LEA in both FDS Phases, representing prevalence rates of 1.0 %, and 1.2 % in FDS2 vs FDS1, respectively (*P* = 0.72). The age-, sex- and ethnicity-adjusted difference between the Phases for LEA prevalence was also not significant [difference (95 % confidence interval) −0.2 (−0.6 to 0.9)  %, *P* = 0.22]. There was a non-significant trend towards fewer major LEAs in FDS2 vs FDS1 (*P* = 0.07) but no difference in the prevalence of minor LEAs (*P* = 0.90). No patient presented with both a minor and major LEA in either Phase.

In multiple logistic regression analysis, independent associates of a baseline LEA in FDS1 were a history of vascular bypass surgery or revascularisation, urinary albumin:creatinine ratio, PSN and cerebrovascular disease (see Table 2). In FDS2, prevalent LEA was also independently associated with a history of vascular bypass surgery or revascularisation, but also past hospitalisation for foot ulcer/current foot ulcer and the fasting serum glucose (see Table 2).

**Table 2:** Independent associates of diabetes-related lower extremity amputation at baseline in Fremantle Diabetes Study Phase 1 (FDS1) and 2 (FDS2). Odds ratios and 95 % confidence intervals (CI) are shown

	FDS1		FDS2	
	Odds ratio (95 % CI)	*P*-value	Odds ratio (95 % CI)	*P*-value
History or peripheral bypass or revascularisation	40.15 (8.02–201.09)	<0.001	33.50 (6.93–161.86)	<0.001
Past hospitalisation for/current foot ulcer			91.65 (19.40–433.08)	<0.001
Fasting serum glucose (increase of 1 mmol/L)			1.36 (1.14–1.62)	0.001
Ln (urinary albumin:creatinine ratio (mg/mmol))	2.04 (1.25–3.32)	0.004		
Cerebrovascular disease	6.11 (1.28–29.16)	0.023		
Peripheral sensory neuropathy	7.52 (1.16–49.01)	0.035		

When FDS1 and FDS2 data were combined, those with LEA at baseline were diagnosed at a younger age and had a longer duration of diabetes than those without LEA, were less likely to be in paid employment, and a greater proportion were Aboriginal (see Table 3). Patients with a LEA were more likely to be treated with insulin (with or without oral blood glucose-lowering agents), but their total serum cholesterol was lower. They had a higher urine albumin:creatinine ratio and were more likely to have an eGFR <60 mL/min/1.73 m^2^, consistent with more frequent micro- and macrovascular complications including retinopathy, PSN, ischaemic heart disease, cerebrovascular disease, PAD, intermittent claudication, as well as past hospitalisation for, or current, foot ulcer, and a history of vascular bypass surgery and/or peripheral revascularisation.

**Table 3:** Characteristics of pooled Fremantle Diabetes Study Phase 1 (FDS1) and 2 (FDS2) participants with or without a diabetes-related lower extremity amputation at baseline. Data are proportions, mean ± SD, geometric mean (SD range), median [IQR] or mean difference (95 % CI)

	No amputation	Amputation	P-value
Number	2775	30	
Age (years)	64.8 ± 11.5	66.4 ± 10.5	0.45
Sex (% male)	50.2	66.7	0.097
Ethnic background (%)			0.020
Anglo-Celt	56.8	46.7	
Southern European	15.1	16.7	
Other European	7.9	10.0	
Asian	3.9	0.0	
Mixed/other	12.0	6.7	
Aboriginal	4.3	20.0	
Age at diabetes diagnosis (years)	56.7 ± 12.0	50.5 ± 16.1	0.005
Duration of diabetes (years)	5.0 (1.75–13.0)	12.0 (9.0–28.0)	<0.001
Education beyond primary level (%)	80.8	78.6	0.81
Paid employment (%)	24.9	7.1	0.027
Married/de facto relationship (%)	64.2	50.0	0.13
Alcohol use (standard drinks/day)	0.11 (0.0–0.75)	0.0 (0.0–1.13)	0.65
Smoking status (%)			0.093
Never	45.2	26.7	
Ex	41.9	63.3	
Current	12.7	10.0	
BMI (kg/m^2^)	30.5 ± 5.9	28.8 ± 5.3	0.14
Obese by waist circumference (%)^a^	67.9	69.6	1.0
Overweight/obese by waist:hip ratio (%)^b^	78.7	81.8	1.0
Fasting serum glucose (mmol/L)	7.5 (6.2–9.6)	7.6 (6.0–10.9)	0.83
HbA1c (%)	7.0 (6.2–8.1)	7.1 (6.5–8.6)	0.40
HbA1c (mmol/mol)	53 (44–65)	54 (48–70)	
Diabetes treatment (%)			<0.001
Diet	28.2	10.0	
Oral agents	54.8	33.3	
Insulin ± oral agents	17.0	56.7	
Systolic blood pressure (mmHg)	148 ± 23	154 ± 33	0.33
Diastolic blood pressure (mmHg)	80 ± 12	79 ± 13	0.61
On antihypertensive therapy (%)	68 ± 19	75 ± 25	0.047
On renin-angiotensin blockers (%)	16.9	23.3	0.33
Total serum cholesterol (mmol/L)	4.9 ± 1.2	4.4 ± 1.2	0.034
Serum HDL cholesterol (mmol/L)	1.16 ± 0.34	1.11 ± 0.32	0.46
Serum triglycerides (mmol/L)	1.8 (1.0–3.2)	1.8 (1.1–2.9)	0.74
Urinary albumin:creatinine ratio	4.0 (1.1–14.7)	23.3 (4.5–121.6)	<0.001
On lipid modifying treatment (%)	41.1	46.7	0.58
Taking aspirin (%)	29.7	40.0	0.23
eGFR <60 mL/min/1.73 m^2^ (%)	19.8	46.7	0.001
Any retinopathy (%)	19.2	53.8	<0.001
Peripheral sensory neuropathy (%)	45.5	82.6	<0.001
History of ischaemic heart disease (%)	28.4	46.7	0.040
History of cerebrovascular disease (%)	8.9	36.7	<0.001
Peripheral arterial disease (%)	24.9	100.0	<0.001
Self-reported intermittent claudication (%)	11.2	33.3	0.001
Arterial bypass/revascularisation (%)	1.7	56.7	<0.001
Past hospitalisation for/current foot ulcer (%)	1.7	53.3	<0.001

^a^Waist circumference ≥ 102.0 cm males, ≥ 88.0 cm females

^b^Waist:hip ratio ≥ 0.95 males, ≥ 0.80 females

In multiple logistic regression analysis, the independent associates of amputation at baseline in the pooled FDS1 and FDS2 datasets included a history of vascular bypass or revascularisation, past hospitalisation for foot ulcer or a foot ulcer at baseline, and higher urinary albumin:creatinine ratio. After adjusting for these variables in the most parsimonious model and then adding FDS Phase as a binary independent variable, Phase 2 was associated with significantly lower risk of a diabetes-related amputation at study entry [odds ratio (95 % confidence interval): 0.28 (0.09–0.84), *P* = 0.023; Table 4].

**Table 4:** Independent associates of diabetes-related lower extremity amputation at baseline in pooled Fremantle Diabetes Study Phase 1 (FDS1) and 2 (FDS2) samples

	Odds ratio (95 % CI)	*P*-value
Ln (urinary albumin:creatinine ratio)	1.74 (1.31–2.32)	<0.001
History of vascular bypass or revascularisation	24.57 (8.22–73.48)	<0.001
Past hospitalisation for/current foot ulcer	23.64 (7.80–71.70)	<0.001
FDS Phase 2	0.28 (0.09–0.84)	0.023

Odds ratios and 95 % confidence intervals (CI) are shown. After adjusting for the most parsimonious model, FDS Phase was added

## Discussion

The key finding in the present study is that the risk of prevalent amputation in two community-based cohorts of patients with type 2 diabetes from the same urban Australian postcode-defined area fell by 72 % over a 15-year period after adjustment for important between-group differences in diabetes-related and other variables. This observation is consistent with recent analyses of large US [3, 11] and Australian [27, 28] population databases, although there was limited access to risk factor and other data in each of these four studies. Because the present substantial reduction in LEA prevalence was independent of improvements in medical and surgical management of diabetes and cardiovascular disease in FDS2 compared with FDS1 patients, there is a strong implication that other factors, including the introduction of government-funded access to regular podiatry services between FDS Phases [29] and the increased availability of high risk foot clinics in public hospitals, were responsible. Indeed, a UK primary care study in 1998 comparing intensive foot care (including more frequent podiatry and associated services) with usual care over 2 years found a similar 70 % reduction in amputation rates [30], while a second UK study also showed a substantial fall in diabetes-related LEAs subsequent to improved organization of diabetes-related foot care [6].

### Epidemiological context

The residents of the FDS catchment area appear representative of the general Australian population. Socio-economic data relating to income, employment, housing, transportation and a range of other variables collected in the 2006 Australian census (which was conducted between FDS1 and FDS2) for the FDS catchment area show an average Index of Relative Socio-economic Advantage and Disadvantage [31] of 1033 with a range by postcode of 977-1113, figures similar to the national mean ± SD which are set at 1000 ± 100. In relation to diabetes-related foot health, this became a national health priority in 1998 [32], 2 years after FDS1 recruitment had closed. Access to diabetes-related allied health services in Australia has been largely through government-funded care plans in which primary care practitioners can refer patients for up to five appointments per year. Foot care is an integral part of initial general diabetes education which is available under this scheme, and there has been a progressive increase in podiatry referrals since the scheme was introduced in 2004 [29]. Australian national recommendations, which were published in 2011, include at least annual pedal examination for all people with diabetes [33]. These considerations suggest that the present findings are generalizable to the Australian population and that they reflect intensification of government-supported foot health initiatives.

A recent assessment of available data has suggested that diabetes-related LEAs have increased 30 % in Australia over approximately a decade since the late 1990s [34]. Although this conclusion was based on disparate data sources without patient-level data, including whether individual patients had multiple hospitalisations for LEA [35, 36], the approximate doubling of diabetes prevalence over the same time period [18, 35] would be expected to increase hospitalisations for LEA on its own. Our data suggest that, although the burden of diabetes is increasing, management strategies that prevent diabetic foot disease have been successful since the FDS1 recruitment period in the 1990s, thus attenuating the risk of LEA even though absolute numbers are increasing.

### Risk factors for amputation and pathophysiological considerations

In the FDS2 and pooled multivariable models, we found that past hospitalisation for foot ulcer or current foot ulceration was strongly and independently associated with prevalent LEA, consistent with the results of previous studies [20, 37, 38]. The FDS1 model included PSN but not past/current foot ulcer, but we have shown that these two variables are tightly linked in FDS1 patients with type 2 diabetes [26]. In addition, vascular bypass surgery or other peripheral revascularisation procedures were strongly and positively associated in individual and pooled models, almost certainly reflecting confounding by indication, while all patients with a history of LEA had PAD, in accord with other studies of risk factors for diabetes-related LEAs [20, 37, 39, 40].

There was a non-significant trend to a reduction in the number of major LEAs from Phase 1 to 2. The results of European-based retrospective studies investigating changes in major LEA amputation rates have been inconsistent. For example, significant reductions in diabetes-related major LEA rates have been found in Denmark and one UK centre [8, 41], non-significant reductions have been reported in England as a whole [42], and non-significant increases have been documented in Ireland [14]. These differences may reflect study-specific differences in case definition (e.g. above the ankle versus above the tarsometatarsal joint) and in the frequency of vascular surgery and other revascularisation procedures. Recent US retrospective data on endovascular surgical procedures and general population major LEA rates between 1996 and 2006 showed a threefold increase in endovascular surgery, a 40 % reduction in bypass surgery, and a 30 % reduction in LEA [43]. The authors suggested that, although endovascular interventions for PAD are being performed more often, a causative link to a reduction in amputations cannot be established on available data given selection and other potential biases.

We identified urinary albumin:creatinine ratio, but not eGFR, as an independent associate of LEA in the multivariable models involving FDS1 and pooled data, consistent with previous studies reporting albuminuria as an important risk factor [20, 44, 45]. Other studies have shown that a low eGFR and end-stage renal disease are associated with diabetes-related LEA [10, 46], but albuminuria was not available for inclusion as an independent variable in the multivariable models used. In any case, patients with renal impairment are likely to have other microvascular and macrovascular complications of diabetes such as PSN and PAD [47] which are strongly associated with foot ulceration [26], thus contributing indirectly to LEA risk.

Given that the substantial reduction in LEA prevalence between FDS Phases was independent of improved diabetes and cardiovascular management, the question arises as to which factors changed with Australian government initiatives to improve foot care in diabetes [29, 32]. One possibility relates to increased surveillance for ulceration and infection. Local factors such as wound depth, severity and presence of infection increase LEA risk [48–50], and early detection of these aspects of foot ulceration would be facilitated by primary care initiatives such as care plans [29]. In addition, availability of advice on other preventive podiatric measures (such as appropriate footwear and strategies to offload pressure points) and regular vascular assessments with a view to surgical or other intervention, may well have increased between FDS Phases through developments such as increased referrals to public hospital high risk foot clinics without being captured in the present multivariable analyses.

Recent studies have assessed potential pathophysiological mechanisms underlying diabetes-associated LEA. In one, there was evidence that increased below-knee arterial calcification scores in patients with type 2 diabetes and normal renal function or mild renal impairment were independently associated with plasma concentrations of dephospho-uncarboxylated matrix Gla protein, a marker of vitamin K status [51]. Although there is preliminary evidence that vitamin K supplementation may reduce vascular calcification, especially in patients with chronic kidney disease [52], whether this will influence rates of LEA complicating diabetes is unknown. There is also evidence that increased circulating concentrations of advanced glycation end products are associated with diabetes-related LEA [53], but this association is attenuated after adjustment for conventional cardiovascular risk factors.

### Limitations of the present study

The FDS patients in both Phases may have included relatively healthy patients but the unadjusted prevalence of diabetes-related LEA in FDS1 (1.2 %) was very close to the 1.3 % prevalence reported in two UK community-based studies conducted between 1988 and 1996 [54, 55]. As previously acknowledged [26], it is possible that there was a change in aspects of data collection requiring subjective assessment, although we maintained standardised procedures for assessment of relevant complications such as PAD and PSN. We did not have measures of plantar foot pressures, joint range of motion, adequacy of footwear or peripheral tissue oxygenation, factors that have been identified as prognostically important in previous studies of diabetes-related foot health [39, 56, 57]. In addition, we did not have complete data on use of public/private podiatry services or attendances at high risk foot clinics to assess whether changes in these variables contributed independently to the decline in diabetes-related LEA between Phases. There were limited numbers of LEAs but the significant independent associates were all clinically plausible. The strengths of the present study include its prospective design, relatively large patient numbers and detailed baseline assessments in each Phase.

## Conclusions

The present study has shown that the risk of prevalent LEA in two cohorts of patients with type 2 diabetes from the same urban Australian community fell substantially over a 15-year period after adjustment for important between-group differences in diabetes-related and other variables. Given its independence from better management of diabetes and cardiovascular disease in FDS2 versus FDS1, this improvement likely reflects the effects of government-funded initiatives to increase awareness of, and access to, services focussed on diabetes-related foot health that were implemented between the two Phases. Given the increasing incidence of diabetes and thus burden of disease including complications such as LEA, this should have clear benefits at both an individual and societal level.

## Abbreviations

ABI: ankle brachial index
FDS: Fremantle Diabetes Study
IQR: interquartile range
LEA: lower extremity amputation
MNSI: Michigan neuropathy screening instrument
PAD: peripheral arterial disease
PSN: peripheral sensory neuropathy
WA: Western Australia
